# Warning of Immortal Time Bias When Studying Drug Safety in Pregnancy: Application to Late Use of Antibiotics and Preterm Delivery

**DOI:** 10.3390/ijerph17186465

**Published:** 2020-09-05

**Authors:** Giovanni Corrao, Federico Rea, Matteo Franchi, Benedetta Beccalli, Anna Locatelli, Anna Cantarutti

**Affiliations:** 1 National Centre for Healthcare Research and Pharmacoepidemiology, Department of Statistics and Quantitative Methods, University of Milano-Bicocca, 20126 Milan, Italy; giovanni.corrao@unimib.it (G.C.); federico.rea@unimib.it (F.R.); matteo.franchi@unimib.it (M.F.); 2 Unit of Biostatistics, Epidemiology and Public Health, Department of Statistics and Quantitative Methods, University of Milano-Bicocca, 20126 Milan, Italy; b.beccalli@campus.unimib.it; 3 Department of Mother and Child, ASST Vimercate, 20871 Vimercate, Italy; anna.locatelli@unimib.it; 4 School of Medicine and Surgery, University of Milano Bicocca, 20900 Monza, Italy

**Keywords:** healthcare use database, pregnancy, antibiotics, preterm birth, immortal time bias

## Abstract

This study aimed to illustrate and account for immortal time bias in pregnancy observational investigations, using the relationship between late use of antibiotics and risk of preterm birth as an example. We conducted a population-based cohort study including 549,082 deliveries between 2007 and 2017 in Lombardy, Italy. We evaluated the risk of preterm births, low birth weight, small for gestational age, and low Apgar score associated with antibiotic dispensing during the third trimester of pregnancy. Cox proportional hazards models were used to estimate hazard ratios (HRs) and 95% confidence intervals (CI) of the outcomes, considering the use of antibiotics as time-fixed (with biased classification of exposure person-time) and time-varying (with proper classification of exposure person-time) exposure. There were 23,638 (4.3%) premature deliveries. There was no association between time-fixed exposure to antibiotics and preterm delivery (adjusted HR 0.96; 95% CI 0.92 to 1.01) but an increased risk of preterm birth when time-varying exposure to antibiotics was considered (1.27; 1.21 to 1.34). The same trend was found for low birth weight and low Apgar score. Immortal time bias is a common and sneaky trap in observational studies involving exposure in late pregnancy. This bias could be easily avoided with suitable design and analysis.

## 1. Introduction

Preterm delivery is estimated to affect 10.6% of all live births around the world, equating to about 15 million births each year [1]. Although there is general consensus on the role of intra-amniotic infection as the main cause of preterm births [2,3,4,5,6,7], there is currently no evidence supporting antibiotic prophylaxis for reducing the risk of preterm delivery and other maternal and newborn adverse outcomes due to infection [8,9]. Rather, as antibiotics are prescribed mostly as therapy, their use can be thought of as a proxy for the onset of some infection; an increased risk in preterm births among women undergoing treatment with antibiotics is therefore expected. At most, the increased risk may be mitigated by treatment with antibiotics, but confidence in this effect is limited given the low certainty of the evidence [10]. Finally, antibiotics are unlikely to be of benefit if they are used late in pregnancy, when inflammatory tissue damage may have already occurred. Accordingly, concern has been expressed that antibiotics may do more harm than good under these circumstances [11,12,13,14,15,16,17].

Nevertheless, no association between third trimester exposure to macrolide antibiotics and the risk of preterm delivery or related outcomes (perinatal mortality, low birth weight, low Apgar score) was reported by a large observational study [18], raising uncertainty about the use of antibiotics in late pregnancy. We suspect that the lack of association may be the result of the improper classification of exposure during follow-up in the study in question. We use this simple observation as an example to illustrate one of the sneakiest traps affecting observational studies in the field of pregnancy investigations.

**Figure 1:**
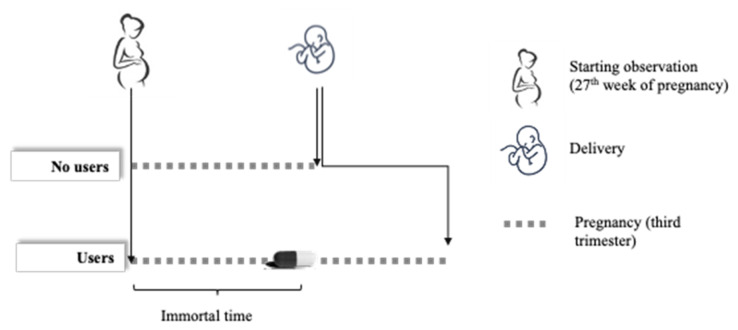
Schematic representation of immortal time bias. From a time-fixed perspective, a woman who did not and another who did use antibiotics during the third trimester of pregnancy are represented. Among users, however, the period between the start of the trimester and the prescribing of an antibiotic should be defined as “immortal,” because it is not possible, by design, for a preterm delivery to occur during this period.

Imagine following a cohort of women from the start of the third trimester of pregnancy (week 27) until delivery and comparing the prevalence of preterm births (i.e., within week 37) between those who use antibiotics and those who do not use them during this period (Figure 1). According to the above-reported premise, a higher risk of preterm births is expected among women who use antibiotics compared with those who do not. However, it should be emphasized that the above-described cohort design may result in biased associations. Consider a woman classified as exposed because an antibiotic is prescribed to her on a certain date. If a preterm birth had occurred before this date, the woman would have been classified as not exposed [19]. Since it is not possible for the outcome to occur before starting exposure, this implies that the time window between the start of the third trimester of pregnancy and the date of first prescription of antibiotics should be defined as “immortal” (i.e., misclassified as an exposure period [20]). This may result in biased estimates, leading to an apparent protective effect of the factor under study.

The origin and implications of immortal time bias in the field of drug safety in pregnancy are reviewed in this paper. Data for a large cohort of women using antibiotics during the third trimester of pregnancy and their risk of adverse events (i.e., preterm birth, low birth weight, small for gestational age (SGA), and low Apgar score) are used to illustrate how immortal time bias can be avoided using appropriate statistical tools and techniques.

## 2. Materials and Methods

### 2.1. Data Sources

The data used for this study were retrieved from the healthcare use databases of Lombardy, a region of Italy that accounts for about 16% (almost ten million) of the national population. All Italian citizens have equal access to healthcare services as part of the National Health Service (NHS); in Lombardy, this is associated with an automated system of databases to collect a variety of information on residents who receive NHS assistance (NHS beneficiaries), diagnoses, procedures performed on inpatients in public or private hospitals (coded according to the International Classification of Diseases, Ninth Revision, Clinical Modification ICD-9-CM codes), outpatient drugs dispensed in community pharmacies (coded according to the Anatomical Therapeutic Chemical (ATC) codes), and specialist visits and diagnostic examinations reimbursable by the NHS. In addition, a database reporting the Certificates of Delivery Assistance (CeDAP) provides detailed information on the mother’s socioeconomic traits, as well as medical information on the pregnancy, childbirth, and child presentation at delivery. As a unique identification code is systematically used for all databases, record linkage provides a large and unselected birth cohort and enables the establishment of relevant traits of and care pathways for mothers and newborns. To preserve privacy, each identification code was automatically deidentified, the inverse process being allowed only to the Regional Health Authority on request from judicial authorities. Diagnostic, therapeutic, and procedural codes used for the current study are given in the Supplementary Material (Tables S1 and S2).

### 2.2. Basic Design

**Figure 2:**
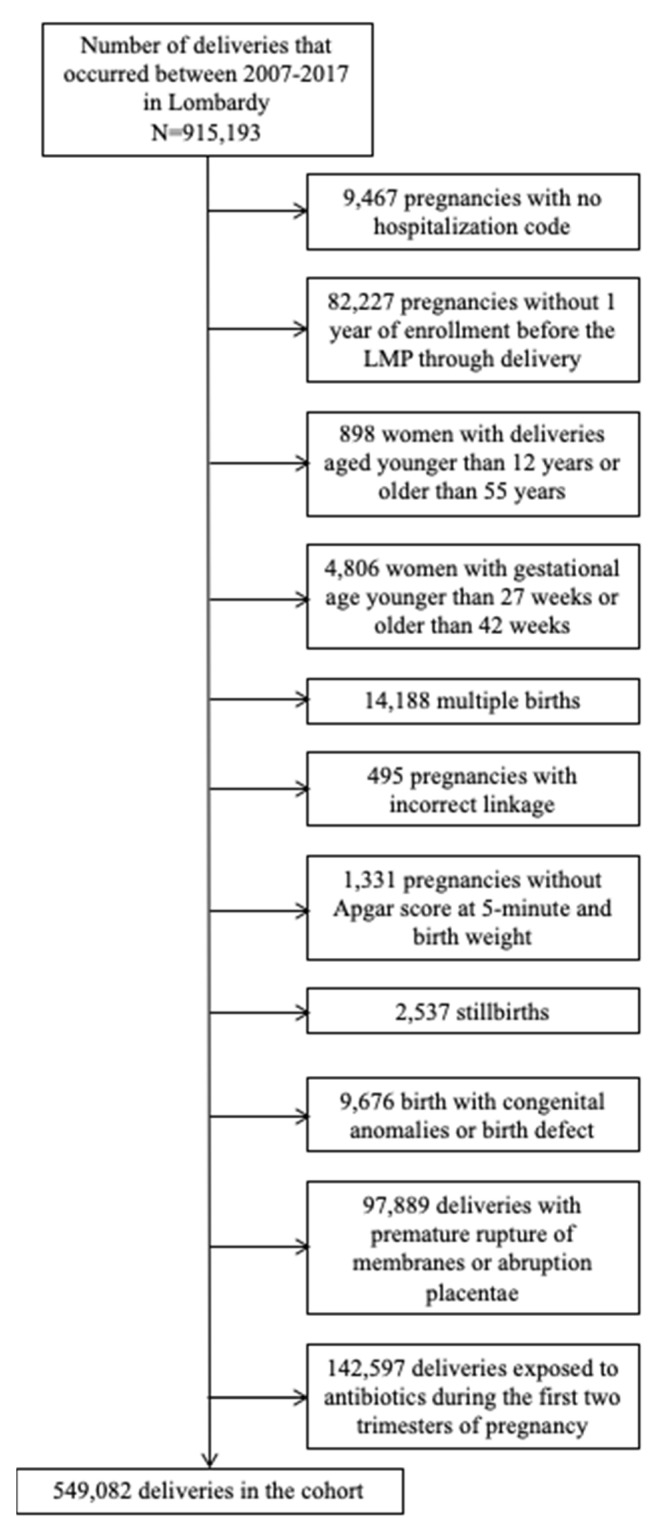
Flowchart of inclusion and exclusion criteria.

The criteria for selecting the study cohort almost completely overlapped with those previously reported by our group [21,22,23]. Briefly, all the 909,489 deliveries occurring in Lombardy between 2007 and 2017 involving women who (i) were beneficiaries of NHS and resident in Lombardy, (ii) were aged 12 to 55 years at delivery, and (iii) had 27 to 42 weeks of gestation, were identified from the CeDAP database. Among these, records were excluded where the newborn was part of a multiple birth, had chromosomal abnormalities, was stillborn, or had missing information for Apgar score at 5 min or weight at birth. In addition, deliveries were excluded for which the mother (i) had a hospital ICD-9-CM code different from the one expected for childbirth, (ii) was not a beneficiary of the regional NHS during the period from 1 year before the last menstrual period (LMP) to delivery, (iii) had antibiotics dispensed during the first two trimesters of pregnancy, or (iv) experienced placental abruption or premature rupture of membranes during the current pregnancy. The final study population therefore consisted of 549,082 mother–newborn pairs (Figure 2). Mothers accumulated person-weeks of observation from 27 weeks of gestation until delivery.

Information on dispensed antibiotics was obtained from the outpatient drug-dispensing database. Accordingly, women were first classified as those for whom, starting from 27 weeks of gestation until delivery, antibiotics were dispensed at least once, and those who never received them.

Users and non-users were compared with respect to sociodemographic features (i.e., age at delivery, nationality, marital status, education, and employment). Reproductive history any time before the last menstrual period (LMP)(parity, previous miscarriage) was also considered. Finally, medical morbidities (i.e., diabetes, hypertension, preeclampsia, neuropathic, non-neuropathic, and other pain, obesity or overweight, substance dependence, and infection), concomitant medications (i.e., non-steroidal anti-inflammatory drugs (NSAIDs) and drugs for acid-related disorders), and use of healthcare services (number of hospitalizations and of distinct dispensed drugs, excluding antibiotics) from 1 year before the LMP until the end of the second trimester of pregnancy were considered. Sources were CeDAP, hospital discharge, and outpatient drug-dispensing databases as appropriate. Users and non-users were compared for each of these covariates using the standardized difference.

The principal outcome of interest was preterm birth (less than 37 gestation weeks [24]). Secondary outcomes including low birth weight (less than 2500 g [25]), SGA [26], and low 5 min Apgar score (less than 7 [27]) were identified through the CeDAP registry (Table S3). Incidence rates (i.e., the ratio between the number of mothers who experienced the outcome and the person-weeks accumulated overall during follow-up) among users and non-users were calculated. The corresponding 95% confidence intervals (CI) were calculated assuming a Poisson distribution.

Cox proportional hazard models were fitted to estimate the hazard ratio (HR) with its 95% CI for a given outcome associated with the “fixed-time” exposure to antibiotics. Three types of models were fitted. In the first, unadjusted HRs were estimated from models including only exposure to antibiotics as a covariate. In the second, partially adjusted HRs were estimated from models also including reproductive history, medical morbidities, concomitant medications, and use of healthcare services as covariates. In the third, fully adjusted HRs were estimated from models also including sociodemographic traits. However, as sociodemographic data were missing for some women, only complete records were considered for this analysis (Table S4).

### 2.3. Accounting for Immortal Time Bias

The basic design ignored the actual timing of antibiotic use; thus, person-weeks from the beginning of the third trimester of pregnancy until delivery entirely contributed to exposure classification [19]. This probably resulted in a spuriously low rate of outcomes for this group compared with non-users. This has been accounted for with two different approaches. For each woman, person-weeks were shifted into their true exposure status [28] with the first approach. In this way, person-weeks accumulated from the start of week 27 gestation until the date of first antibiotic dispensing were correctly classified as unexposed person-weeks. By means of this “time-varying” approach, incidence rates during person-weeks covered by use and non-use of antibiotics were calculated, together with the corresponding 95% CI.

A model-based approach was subsequently used. As outcome rates were expected to vary considerably during the considered period, rather than fitting a Poisson regression model (i.e., the more natural way of modeling incidence rates), Cox proportional hazard models were fitted to estimate the HR and 95% CI of a given outcome associated with the “time-varying” exposure to antibiotics. In other words, at each event time (in our case, whenever a preterm birth occurred), prevalence of antibiotic use by mother with less than 37 weeks of gestation was compared with prevalence of antibiotic use during the same time frame by mothers who had not yet given birth at that time [29]. Unadjusted, partially adjusted, and fully adjusted estimates were calculated as described above.

All analyses were performed using the Statistical Analysis System software (version 9.4; SAS Institute, Cary, NC, USA). Statistical significance was set at 0.05, and p-values were two-sided.

## 3. Results

Table 1 compares selected traits of women who used and who did not use antibiotics during the third trimester of pregnancy. Overall, 44,772 (8.1%) women were exposed to antibiotics during the third trimester. Women who used antibiotics were similar to those who did not use them, with the exception of a higher prevalence of unemployment, infection, and NSAID use, as well as use of drugs for acid-related disorders and number of distinct dispensed drugs, excluding antibiotics.

Table 2 reports the biased (time-fixed) and unbiased (time-varying) analyses for the association between the use of antibiotics and selected outcomes. First, we consider preterm birth as the main outcome of interest. In the biased time-fixed analysis, users and non-users had approximately the same rate (about four events every 1000 person-weeks). In this analysis, the entire 380,389 person-weeks of follow-up accumulated by the 38,359 women for whom antibiotics were dispensed during the third trimester were classified as exposed. However, these 380,389 person-weeks included 186,718.57 person-weeks (i.e., the 49% of total follow-up time) of misclassified/immortal unexposed person-time (i.e., the time from the beginning of the trimester until the first dispensed antibiotic). Reclassifying this unexposed person-time by the denominator of the incidence rate of the unexposed group led to varying incidence rates of preterm delivery in the exposed and unexposed groups (nine vs. four events every 1000 person-weeks). Accordingly, the time-varying approach led to a HR suggestive of a 32% excess risk of preterm birth among users compared with non-users (95% CI, 25% to 38%). Although part of the excess risk was explained by the covariates considered, evidence of a risk of preterm birth positively associated with antibiotic use was observed for partially and fully adjusted estimates. The association between antibiotic use and the other considered outcomes systematically changed direction from biased to unbiased estimates. In summary, there were significant positive associations between antibiotics and preterm birth, low birth weight, and low 5 min Apgar score. Conversely, although a significant negative association between antibiotics and SGA was shown by the time-fixed approach, there was no evidence of such an effect in the time-varying analyses.

**Figure 3:**
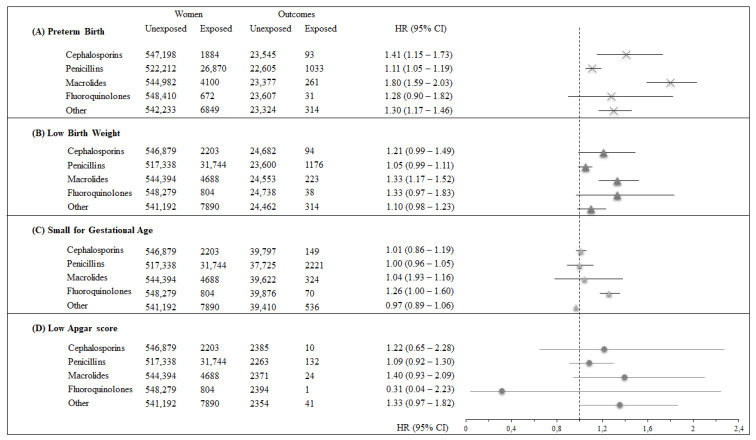
Forest plot for the association between time-varying use of cephalosporin, penicillin, macrolides, fluoroquinolones, and all other antibiotics together, and the risk of preterm birth (box (**A**)), low birth weight (box (**B**)), small for gestational age (box (**C**)), and low 5 min Apgar score (box (**D**)).

Finally, as shown in Figure 3, time-varying exposure to single antibiotic classes (i.e., cephalosporins, penicillins, macrolides, fluoroquinolones, and all others together) increased the risk of preterm birth, low birth weight, and low 5 min Apgar, although power concern prevented some of these associations from reaching conventional levels of significance.

## 4. Discussion

Immortal time bias is one of the sneakiest traps affecting observational studies in the field of pregnancy investigations. We showed how this bias might act by investigating the relationship between use of antibiotics during the third trimester of pregnancy and preterm birth and its correlates linked with neonatal health (birth weight and Apgar score). The rationale for using this example is as follows. Readers may verify that when investigating the association of interest by means of a conventional cohort study, i.e., by comparing users and non-users of antibiotics with respect to the occurrence of preterm birth, an unexpected independence or even protective action of antibiotics is systematically observed. This is quite strange, as use of antibiotics is thought of as a proxy for the onset of some kind of infection, which is one of the major causes of preterm births [4,5,6,7,8,9]. The present study showed how incorrect allocation of exposure person-time may strongly affect and even reverse the direction of the investigated association. In practice, as women for whom antibiotics were dispensed spent part of their follow-up without antibiotic exposure, and because this period must be considered immortal (that is, the event cannot occur, otherwise the woman would have been classified as unexposed), person-weeks spent with no use should be suitably allocated in order to obtain unbiased estimates.

The limitations of our study include those that conventionally affect observational investigations based on healthcare use databases. It should, however, be emphasized that because antibiotic dispensing was assumed to be a surrogate for the occurrence of infection, the usual concern about the uncertainty of patients’ compliance with the drug treatment regimen should not be of interest in our example. Rather, the delay between infection onset and the date of drug dispensing could lead to misclassification of a certain number of person-weeks as currently exposed to infection, thus diluting the point estimates toward the null. In addition, antibiotics dispensed over the counter might also drag our estimates toward the null. Finally, as for all observational studies, there is some concern regarding residual confounding due to unmeasured factors. Some lifestyle factors, as well as drugs dispensed during hospitalization and the over the counter medications, are not recorded in our database, resulting in a loss of information. Therefore, because some drugs associated with reduced preterm delivery rates (e.g., progesterone, tocolytic drugs, and antenatal steroids) were not taken into account in our study, further investigations are needed to better understand the causal model of preterm birth and its correlates.

## 5. Conclusions

In conclusion, we described immortal time bias as a common and sneaky trap affecting observational studies investigating exposure during pregnancy and preterm births, or other outcomes associated with preterm birth. We showed that this bias could be easily avoided with suitable design and analysis.

## Figures and Tables

**Table 1:** Selected characteristics of women who used and who did not use antibiotics during the third trimester of pregnancy.

	Antibiotics		Standardized Difference
Mothers’ Characteristics	Users	Non-Users	
	(*n* = 44,772)	(*n* = 504,310)	
**Sociodemographic Traits**			
Maternal age, mean (SD), years	32.1 (5.31)	32.3 (5.10)	−0.04
Nationality			
Italian	32,190 (71.9%)	376,316 (74.6%)	−0.06
Other	11,074 (24.7%)	112,111 (22.2%)	0.06
Unknown	1508 (3.4%)	15,883 (3.2%)	0.01
Education			
Low	13,370 (29.9%)	124,837 (24.8%)	0.11
Intermediate	19,628 (43.8%)	224,856 (44.6%)	−0.01
High	11,450 (25.6%)	151,544 (30.1%)	−0.10
Unknown	324 (0.7%)	3073 (0.6%)	0.01
Marital status			
Married	30,909 (69.0%)	345,199 (68.5%)	0.02
Unmarried	13,185 (29.5%)	152,742 (30.3%)	−0.01
Unknown	678 (1.5%)	6369 (1.3%)	0.02
Occupation			
Employed	30,324 (67.7%)	367,642 (72.9%)	−0.11
Unemployed	14,295 (31.9%)	135,223 (26.8%)	0.11
Unknown	153 (0.3%)	144 (0.3%)	0.01
**Reproductive History and Status**			
Primiparous	12,689 (28.3%)	162,008 (32.1%)	−0.07
Previous miscarriage	11,523 (25.7%)	121,136 (24.0%)	0.04
Gestational age, mean (SD%), weeks	39.1 (1.4%)	39.1 (1.6%)	−0.01
**Medical Conditions**			
Substance dependence	24 (0.1%)	150 (0.0%)	0.01
Infection	528 (1.2%)	3,859 (0.8%)	0.04
Hypertension	98 (0.2%)	1,283 (0.3%)	−0.01
Preeclampsia	42 (0.1%)	555 (0.1%)	−0.01
Diabetes	172 (0.4%)	1,843 (0.4%)	0.00
Obesity or overweight	66 (0.2%)	356 (0.1%)	0.02
Dyslipidemia	7 (0.0%)	41 (0.0%)	0.01
Neuropathic, non-neuropathic, and other pain	212 (0.5%)	1986 (0.4%)	0.01
C-section	12,451 (27.8%)	140,185 (27.8%)	0.00
**Medications**			
NSAIDs	2823 (6.3%)	22,698 (4.5%)	0.08
Drugs for acid-related disorders	4733 (10.6%)	39,112 (7.8%)	0.10
**Use of Healthcare Services**			
Hospitalizations	10,014 (22.4%)	104,134 (20.7%)	0.04
No. of distinct dispensed drugs, excluding antibiotics			
=1	14,296 (31.9%)	158,239 (31.4%)	0.01
≥2	14,854 (33.2%)	130,506 (25.9%)	0.16

NSAIDs, non-steroidal anti-inflammatory drugs.

**Table 2:** Biased (time-fixed) and unbiased (time-varying) approaches for the association between antibiotic use during the third trimester of pregnancy and selected outcomes (preterm birth, low birth weight, small for gestational age, and low Apgar score at 5 min.).

		No. of Women	No. of Events	Person-Weeks	Rate	Crude		Partially Adjusted		Fully Adjusted	
					(Per 1000 Weeks)	HR	(95% CI)	HR	(95% CI)	HR	(95% CI)
Preterm birth	**Biased Approach**										
	Non-users	510,723	21,987	5,054,083	4	1.00	(reference)	1.00	(reference)	1.00	(reference)
	Users	38,359	1651	380,389	4	1.00	(0.95–1.05)	0.96	(0.92–1.01)	0.95	(0.90–1.00)
	**Unbiased Approach**										
	Non-users	510,723	21,987	5,240,801.57	4	1.00	(reference)	1.00	(reference)	1.00	(reference)
	Users	38,359	1651	193,670.43	9	1.32	(1.25–1.38)	1.27	(1.21–1.34)	1.25	(1.19–1.32)
Low birth weight	**Biased Approach**										
	Non-users	504,310	23,019	6,089,254	4	1.00	(reference)	1.00	(reference)	1.00	(reference)
	Users	44,772	1757	543,276	3	0.85	(0.81–0.90)	0.83	(0.79–0.87)	0.82	(0.78–0.86)
	**Unbiased Approach**										
	Non-users	504,310	23,019	6,347,983.86	4	1.00	(reference)	1.00	(reference)	1.00	(reference)
	Users	44,772	1757	284,546.14	6	1.14	(1.09–1.20)	1.12	(1.06–1.17)	1.10	(1.05–1.16)
Small for gestational age	**Biased Approach**										
	Non-users	504,310	36,836	608,9254	6	1.00	(reference)	1.00	(reference)	1.00	(reference)
	Users	44,772	3110	543,276	6	0.94	(0.90–0.97)	0.93	(0.90–0.96)	0.92	(0.89–0.96)
	**Unbiased Approach**										
	Non-users	504,310	36,836	6,347,983.86	6	1.00	(reference)	1.00	(reference)	1.00	(reference)
	Users	44,772	3110	284,546.14	11	1.01	(0.98–1.05)	1.00	(0.97–1.04)	0.99	(0.96–1.03)
Low Apgar at 5 min	**Biased Approach**										
	Non-users	504,310	2196	6,089,254	0.4	1.00	(reference)	1.00	(reference)	1.00	(reference)
	Users	44,772	199	543,276	0.4	1.01	(0.87–1.16)	0.98	(0.85–1.14)	0.96	(0.83–1.12)
	**Unbiased Approach**										
	Non-users	504,310	2196	6,347,983.86	0.3	1.00	(reference)	1.00	(reference)	1.00	(reference)
	Users	44,772	199	284,546.14	1	1.23	(1.06–1.42)	1.20	(1.04–1.39)	1.17	(1.01–1.36)

HR, hazard ratios; CI, confidence intervals.

## Supplementary Materials

The following are available online at https://www.mdpi.com/1660-4601/17/18/6465/s1, Table S1: Antibiotic Therapy, Table S2: ICD-9 Diagnostic codes used to identify neonatal outcomes, Table S3: Distribution of neonatal outcomes, Table S4: Missing percentage among the selected cohort. Lombardy region, 2007–2017.

## References

[1] Chawanpaiboon S., Vogel J.P., Moller A.-B., Lumbiganon P., Petzold M., Hogan D., Landoulsi S., Jampathong N., Kongwattanakul K., Laopaiboon M. (2018). Global, regional, and national estimates of levels of preterm birth in 2014: A systematic review and modelling analysis. Lancet Glob. Heal..

[2] Costeloe K.L., Hennessy E., Gibson A.T., Marlow N., Wilkinson A.R., EPICure Study Group (2000). The EPICure Study: Outcomes to Discharge From Hospital for Infants Born at the Threshold of Viability. Pediatrics.

[3] Petrou S., Abangma G., Johnson S.J., Wolke D., Marlow N. (2009). Costs and Health Utilities Associated with Extremely Preterm Birth: Evidence from the EPICure Study. Value Heal..

[4] Morency A.-M., Bujold E. (2007). The effect of second-trimester antibiotic therapy on the rate of preterm birth. J. Obstet. Gynaecol. Can..

[5] Yoon B.H., Romero R., Bin Moon J., Shim S.-S., Kim M., Kim G., Jun J.K. (2001). Clinical significance of intra-amniotic inflammation in patients with preterm labor and intact membranes. Am. J. Obstet. Gynecol..

[6] Jacobsson B., Mattsby-Baltzer I., Andersch B., Bokstrom H., Holst R.-M., Wennerholm U.-B., Hagberg H. (2003). Microbial invasion and cytokine response in amniotic fluid in a Swedish population of women in preterm labor. Acta Obstet. Gynecol. Scand..

[7] Wahbeh C.J., Hill G.B., Eden R.D., Gall S.A. (1984). Intra-amniotic bacterial colonization in premature labor. Am. J. Obstet. Gynecol..

[8] Greig P.C. (1998). The Diagnosis of Intrauterine Infection in Women with Preterm Premature Rupture of the Membranes (PPROM). Clin. Obstet. Gynecol..

[9] Watts H.D., Krohn M.A., Hillier S.L., Eschenbach D.A. (1992). The Association of Occult Amniotic Fluid Infection with Gestational Age and Neonatal Outcome Among Women in Preterm Labor. Obstet. Gynecol..

[10] Kenyon S., Taylor D., Tarnow-Mordi W. (2001). Broad-spectrum antibiotics for spontaneous preterm labour: The ORACLE II randomised trial. Lancet.

[11] Thinkhamrop J., Hofmeyr G.J., Adetoro O., Lumbiganon P., Ota E. (2015). Antibiotic prophylaxis during the second and third trimester to reduce adverse pregnancy outcomes and morbidity. Cochrane Database Syst Rev..

[12] Smaill F.M., Vazquez J.C. (2019). Antibiotics for asymptomatic bacteriuria in pregnancy. Cochrane Database Syst. Rev..

[13] Lamont R.F. (1998). Infection and preterm labour. BJOG Int. J. Obstet. Gynaecol..

[14] Lamont R.F. (2000). Antibiotics for the Prevention of Preterm Birth. N. Engl. J. Med..

[15] Lamont R.F. (2009). Association between cerebral palsy and erythromycin. Lancet.

[16] Mason M.R., Adrinkra P.E., Lamont R.F. (2000). Prophylactic administration of clindamycin 2% vaginal cream to reduce the incidence of spontaneous preterm birth in women with an increased risk: A randomised placebo-controlled double-blind trial. BJOG Int. J. Obstet. Gynaecol..

[17] Lamont R.F. (2005). The maternal fetal medicine network trial. Am. J. Obstet. Gynecol..

[18] Dinur A.B., Koren G., Matok I., Wiznitzer A., Uziel E., Gorodischer R., Levy A. (2013). Fetal Safety of Macrolides. Antimicrob. Agents Chemother..

[19] Matok I., Azoulay L., Yin H., Suissa S. (2014). Immortal time bias in observational studies of drug effects in pregnancy. Birth Defects Res. Part A: Clin. Mol. Teratol..

[20] Suissa S. (2003). Effectiveness of Inhaled Corticosteroids in Chronic Obstructive Pulmonary Disease. Am. J. Respir. Crit. Care Med..

[21] Cantarutti A., Franchi M., Rea F., Merlino L., Corrao G. (2018). Use of Nimesulide During Early Pregnancy and the Risk of Congenital Malformations: A Population-Based Study from Italy. Adv. Ther..

[22] Cantarutti A., Merlino L., Giaquinto C., Corrao G. (2017). Use of antidepressant medication in pregnancy and adverse neonatal outcomes: A population-based investigation. Pharmacoepidemiol. Drug Saf..

[23] Cantarutti A., Merlino L., Monzani E., Giaquinto C., Corrao G. (2016). Is the Risk of Preterm Birth and Low Birth Weight Affected by the Use of Antidepressant Agents during Pregnancy? A Population-Based Investigation. PLOS ONE.

[24] Lawn J.E., Gravett M.G., Nunes T.M., Rubens C.E., Stanton C.K. (2010). Global report on preterm birth and stillbirth (1 of 7): Definitions, description of the burden and opportunities to improve data. BMC Pregnancy Childbirth.

[25] De Bernabé J.V., Soriano T., Albaladejo R., Juarranz M., Calle M.E., Martínez D., Domínguez-Rojas V. (2004). Risk factors for low birth weight: A review. Eur. J. Obstet. Gynecol. Reprod. Boil..

[26] Phiri K., Hernandez-Diaz S., Tsen L.C., Puopolo K.M., Seeger J.D., Bateman B.T. (2015). Accuracy of ICD-9-CM coding to identify small for gestational age newborns. Pharmacoepidemiol. Drug Saf..

[27] Casey B.M., McIntire D.D., Leveno K.J. (2001). The Continuing Value of the Apgar Score for the Assessment of Newborn Infants. N. Engl. J. Med..

[28] Lévesque L.E., Hanley J.A., Kezouh A., Suissa S. (2010). Problem of immortal time bias in cohort studies: Example using statins for preventing progression of diabetes. BMJ.

[29] Fisher L.D., Lin D.Y. (1999). Time-dependent covariates in the cox proportional-hazards regression model. Annu. Rev. Public Heal..

