# Supplementary material for: Warning of Immortal Time Bias When Studying Drug Safety in Pregnancy: Application to Late Use of Antibiotics and Preterm Delivery
Source: Int J Environ Res Public Health. 2020 Sep 5;17(18):6465. doi: 10.3390/ijerph17186465 (PMC7558278; doi:10.3390/ijerph17186465)
Supplement: Supplementary file 1 [file ijerph-17-06465-s001.pdf]

Supplementary Appendix

This appendix was provided by the authors to give readers additional information about their work.

Supplement to: Giovanni Corrao, Federico Rea, Matteo Franchi, Benedetta Beccalli, Anna Locatelli, Anna Cantarutti

Figure and Table of Contents

Table S1 Antibiotic Therapy
Table S2 ICD-9 Diagnostic codes used to identify neonatal outcomes
Table S3 Distribution of neonatal outcomes
Table S4 Missing percentage among the selected cohort. Lombardy region, 2007-2017

Table S1. Antibiotic Therapy.

ATC codes	Antibiotic classes
J01A	Tetracyclines
J01B	Amphenicols
J01C	Beta-lactam antibacterials, penicillins
J01D	Other beta-lactam antibacterials
J01E	Sulfonamides and trimethoprim
J01F	Macrolides, lincosamides and streptogramins
J01G	Aminoglycoside antibacterials
J01M	Quinolone antibacterials
J01R	Combinations of antibacterials
J01X	Other antibacterials

ATC, Anatomical Therapeutic Chemical

Table S2. ICD-9 Diagnostic codes used to identify neonatal outcomes.

ICD-9 Diagnosis and Procedure Codes	
Delivery	
ICD-9 Diagnosis	V27.xx; 640.xx – 676.xx
ICD-9 Procedure	73.2x; 73.5x; 73.6; 73.8; 73.9x; 72.xx; 74.0 – 74.2; 74.4
Placenta abruption	
ICD-9 Diagnosis	762.1; 641.2
Premature rupture of membranes	
ICD-9 Diagnosis	658.1; 658.2; 761.1

ICD-9, International Classification of Diseases, Ninth Revision

Table S3. Distribution of neonatal outcomes.

Preterm Birth	Low Birth Weight	Small for Gestational Age	Low Apgar Score at 5 min.	N	(%)
				485,753	(88.47)
				1602	(0.29)
				25,051	(4.56)
				130	(0.02)
				995	(0.18)
				3	(0.00)
				11,824	(2.15)
				86	(0.02)
				11,661	(2.12)
				108	(0.02)
				8652	(1.58)
				361	(0.07)
				2750	(0.50)
				105	(0.02)

Table S4. Missing percentage among the selected cohort. Lombardy region, 2007–2017.

	Missing (%)	
	Users	Non users
Marital status	1.51	1.26
Employment status	0.34	0.29
Educational attainmet	0.72	0.61
Nationality	3.37	3.15
